# Is there an ideal way to initiate antiplatelet therapy with aspirin? A crossover study on healthy volunteers evaluating different dosing schemes with whole blood aggregometry

**DOI:** 10.1186/1756-0500-4-106

**Published:** 2011-04-05

**Authors:** Saskia H Meves, Horst Neubauer, Ursula Overbeck, Heinz G Endres

**Affiliations:** 1 Ruhr University Bochum, Department of Neurology, St. Josef - Hospital, Gudrunstrasse 56, D-44791 Bochum, Germany; 2 Ruhr University Bochum, Cardiovascular Center, St. Josef - Hospital, Gudrunstrasse 56, D-44791 Bochum, Germany; 3 Ruhr University Bochum, D-44780 Bochum, Germany

**Keywords:** Aspirin, Stroke, Loading Dose, Pharmacokinetics, Aggregometry

## Abstract

**Background:**

Guidelines recommend an early initiation of aspirin treatment in patients with acute cerebral ischemia. Comparative studies on the best starting dose for initiating aspirin therapy to achieve a rapid antiplatelet effect do not exist. This study evaluated the platelet inhibitory effect in healthy volunteers by using three different aspirin loading doses to gain a model for initiating antiplatelet treatment in acute strokes patients.

**Methods:**

Using whole blood aggregometry, this study with a prospective, uncontrolled, open, crossover design examined 12 healthy volunteers treated with three different aspirin loading doses: intravenous 500 mg aspirin, oral 500 mg aspirin, and a course of 200 mg aspirin on two subsequent days followed by a five-day course of 100 mg aspirin. Aspirin low response was defined as change of impedance exceeding 0 Ω after stimulation with arachidonic acid.

**Results:**

Sufficient antiplatelet effectiveness was gained within 30 seconds when intravenous 500 mg aspirin was used. The mean time until antiplatelet effect was 74 minutes for 500 mg aspirin taken orally and 662 minutes (11.2 hours) for the dose scheme with 200 mg aspirin with a high inter- and intraindividual variability in those two regimes. Platelet aggregation returned to the baseline range during the wash-out phase within 4 days.

**Conclusion:**

Our study reveals that the antiplatelet effect differs significantly between the three different aspirin starting dosages with a high inter- and intraindividual variability of antiplatelet response in our healthy volunteers. To ensure an early platelet inhibitory effect in acute stroke patients, it could be advantageous to initiate the therapy with an intravenous loading dose of 500 mg aspirin. However, clinical outcome studies must still define the best way to initiate antiplatelet treatment with aspirin.

## Background

Initiating early antiplatelet therapy with aspirin (acetylsalicylic acid, ASA) is important in treating patients with acute ischemic stroke or transient ischemic attacks (TIAs) and has been proven to significantly decrease the overall risk of further strokes or death [1-3]. Based on the results of two large randomized trials with up to 40 000 patients, present guidelines recommend starting aspirin treatment within 48 hours after stroke onset with doses varying from 100 mg to 325 mg to reduce the risk of early stroke recurrence [1-6].

Recommendations for the antiplatelet treatment of coronary or peripheral disease cannot be transferred directly to the treatment of patients with acute strokes. This fact is ascribed to the heterogenic stroke causes and the bleeding vulnerability of the brain after acute cerebrovascular ischemia [7]. Treating acute stroke patients, neurologists commonly feel they are between Scylla and Charybdis as they try to reduce the risk of recurrent thromboembolic events but at the same time fear an excessive risk of cerebral bleeding when using antithrombotic agents. Data of the IST and CAST-study groups showed an insignificant increase of hemorrhagic stroke or transformation with aspirin doses of either 160 mg or 300 mg and no elevated net hazard in patients who had been inadvertently randomized after a hemorrhagic stroke [1-3]. Still higher doses of aspirin could lead to an increase of cerebral hemorrhage in analogy to dose-dependent extracranial bleeding complications. Present-day guideline recommendations on aspirin dosages for acute stroke patients are based on these two major clinical observational studies carried out in the Nineties. At the time these studies were made, there was no routine monitoring of the effectiveness of antiplatelet drugs.

As nowadays bedside testing of the antiplatelet effect has become available using such tests as impedance aggregometry, the antiplatelet effect of aspirin can be monitored and calibrated much more easily in patients with ischemic stroke. Given the limited data on how to initiate aspirin treatment in stroke patients, our study evaluated the dose-time response relationship of different aspirin dosages in healthy volunteers. Using whole blood aggregometry, the effect of three different aspirin dosing regimens was assessed in order to find the best pharmacodynamic way to initiate acute antiplatelet treatment.

## Methods

### Study Population

12 healthy volunteers without any previous medical history or chronic drug therapy were enrolled in this study after informed consent was obtained. The participants did not have any history of bleeding diathesis, drug or alcohol abuse, kidney or liver disease and abstained from any medication two weeks prior to study initiation and during the study protocol. In addition, normal prothrombin time, a normal platelet count (within 150-400 × 10^9^/L), normal liver function (GOT and GPT within the normal range), normal kidney function and normal serum creatinine (<1.2 mg/dl) were mandatory to participate in this study. The study was approved by the local Ethics Committee of the Ruhr-University Bochum.

### Blood Sampling and Intervention

All participants were assigned in a prospective, uncontrolled, open crossover design to three different treatment schemes for initiating the antiplatelet treatment with aspirin (ASPIRIN^®^; Bayer Health Care; Leverkusen, Germany): the first treatment option was aspirin 500 mg given intravenously and contra-laterally to the blood donor's arm (regimen 1), secondly an aspirin 500 mg tablet taken orally (regimen 2) and the third option was an oral dose of 200 mg aspirin for the first two subsequent days, followed by 100 mg aspirin daily for 5 days (regimen 3). The study medication was taken in the presence of a study investigator. The laboratory technician for aggregometry was blinded for the treatment regimen and the aspirin dose used. The wash-out period between each test series lasted more than two weeks.

Blood samples were drawn with an 18 gauge needle from an antecubital vein before and after intake of the initial dose. The first 10 ml of blood were discarded to avoid spontaneous platelet activation after insertion of the needle. Blood was then collected in 4.0 ml blood tubes containing 0.4 ml citrate (KABE Labortechnik, Nümbrecht, Germany). For practical reasons, different time intervals for blood drawing were set in order to reduce the number of blood samples and were ceased when a consistent antiaggregatory effect became apparent (Table 1). Blood samples were taken for each treatment option at baseline, after 5, 10, 15, 30, 45, 60, 120, 180, 240 and 360 minutes (min). Because of the expected rapid-onset effect of 500 mg aspirin, either intravenously (regimen 1) or orally applied (regimen 2), additional measurements were performed after 0.5, 1, 2.5 and 7.5 min. For dosing regimen 3, all measurements were repeated on day 2. To evaluate the time period until the antiplatelet effect of aspirin was no longer detectable, subjects of treatment schemes 2 and 3 were monitored once daily during the wash-out phase after aspirin cessation.

**Table 1:** Overview of the measurement time points

Aspirin Dose (mg)	Time in minutes													
	
	b	0.5	1	2.5	5	10	15	30	45	60	120	180	240	360
**500 i.v**.	x	x	x	x	x	x	x	x	x	x	x	x	x	-

**500 oral**	x	-	x	x	x	x	x	x	x	x	x	x	x	x

**200 oral**	x	-	-	-	x	x	x	x	x	x	x	x	x	x

b indicates basal measurement, i.v. intravenous

### Impedance Aggregometry

Platelet function testing was done by using impedance aggregometry (Model 590, Chrono-log Corporation, Havertown, PA, USA) as previously described [8,9] and the data were analyzed with the AggroLink software package. This system measures the change of electrical impedance in the test cuvette caused by aggregation and adhesion of platelets to the electrode's surface. Impedance aggregometry testing with arachidonic acid and collagen (Chrono-Par, Chrono-log Corporation, Havertown, PA, USA) was performed at the time intervals described above. Measurements were carried out at 37°C and a stirring speed of 1000 rpm between 60 to 180 min after blood collection. 500 μl of citrated blood was diluted 1:1 with sodium chloride 0.9% and heated for at least 5 min at 37°C in a polycarbonate cuvette. After calibration was performed and a stable baseline (steady state) had been established, the two agonists were added to different aliquots and aggregation was monitored for 6 min. As agonists 10 μM arachidonic acid (AA) with a final concentration of 0.5 mM and 2 μl collagen with a final concentration of 2 μg/ml were used and the maximum change of impedance (Ω) as well as the area under the impedance curve (AUC) were recorded. Aspirin low response (insufficient platelet inhibitory response) as the main outcome parameter was defined as a change of impedance exceeding 0 Ω after stimulation with AA in accordance with previous studies and recommendations [9,10]. Accordingly sufficient platelet inhibitory response and effective aspirin responsiveness stands for 0 Ω.

### Statistics

Our study had a 3-period 3-treatment crossover design. All impedance or AUC values for the three treatment regimes were averaged for each measurement time point. Variables are presented as mean ± standard deviation (SD). Differences in means between the two oral aspirin groups were tested for significance using the paired Student's *t *test under the Bonferroni-adjusted significance level of p = 0.0045 (= 0.05/11) for multiple comparisons. Data are presented as mean values (95% confidence interval) for single treatment groups and mean (99.55% confidence interval) when analyzing multiple comparisons of mean differences.

To calculate the mean and standard deviation of ohm and AUC values without the intake of aspirin, 10 measurements of impedance and AUC values at identical time points of regimen 2 from basal (0 min) until 240 min were taken and the coefficient of variation determined. All analyses were performed using SAS, version 9.2 (SAS Institute Inc., Cary, NC, USA). All curves were plotted using SPSS, version 18.0 (SPSS Inc., Chicago, Ill, USA).

## Results

### Baseline Characteristics

12 healthy volunteers (6 male, 6 female; mean age 34.8 ± 9.2 years) participated in this study. Measurements for consistency without the intake of aspirin showed for AA stimulated samples a mean value of 14.1 ± 1.4 Ω for impedance and 64.82 ± 6.0 arbitrary units (AU) for AUC analysis. For collagen stimulation the results were 21.0 ± 1.7 Ω and 80.43 ± 6.5 AU respectively. The results revealed a good reproducibility with a standard deviation of 1.4 Ω and a variation coefficient of 0.10.

There were no significant differences between the three groups at baseline: for treatment regimen 1 (500 mg aspirin i.v.) the values were 15.3 ± 2.1 Ω for stimulation with AA and 21.4 ± 3.5 Ω for collagen induced aggregometry, compared to 13.6 ± 4.4 Ω and 20.1 ± 6.4 Ω for regimen 2 (aspirin 500 mg orally) and for regimen 3 with 200 mg aspirin orally as starting dose 13.2 ± 2.3 Ω and 16.9 ± 4.0 Ω.

On comparing the different time points, mean impedance values after oral intake of either 200 mg or 500 mg aspirin showed no significant differences, yet a wide range of values with high inter- and intraindividual differences were present (Table 2). In contrast, intravenous 500 mg aspirin attained a sufficient platelet inhibitory response of 0 Ω in all 12 volunteers within 30 seconds.

**Table 2:** Mean impedance values (in ohm (Ω)) comparing 200 mg and 500 mg oral and 500 mg intravenous aspirin dosages

	Aspirin 200 mg oral	Aspirin 500 mg oral	**Aspirin 500 mg i.v**.	P-value (200 mg vs. 500 mg oral)
**Day 1 (0 min)**	13.2 (11.7 - 14.6)	13.6 (10.8 - 16.4)	15.3 (14.0 - 16.7)	0.80

**5 min**	12.8 (9.9 - 15.6)	12.8 (10.2 - 15.4)	0.0	0.96

**10 min**	12.3 (9.4 - 15.1)	11.8 (7.6 - 16.1)	0.0	0.87

**15 min**	9.3 (5.4 - 13.3)	8.3 (4.4 - 12.1)	0.2 (-0.4 - +0.8)	0.68

**30 min**	3.8 (0.6 - 6.9)	4.2 (0.5 - 7.8)	0.0	0.87

**45 min**	3.8 (0.4 - 7.3)	2.6 (-0.05 - 5.2)	0.25 (-0.5 - +1.0)	0.52

**60 min**	3.9 (0.06 - 7.8)	1.3 (-0.3 - 2.9)	0.2 (-0.4 - +0.8)	0.17

**120 min**	3.2 (-0.6 - 6.9)	1.3 (-0.6 - 3.1)	0.2 (-0.4 - +0.8)	0.30

**180 min**	1.8 (-0.6 - 4.1)	1.3 (-0.6 - 3.3)	0.0	0.69

**240 min**	2.1 (-0.4 - 4.6)	0.7 (-0.8 - 2.1)	0.2 (-0.4 - +0.8)	0.11

**Day 2 (0 min)**	3.0 (0.6 - 5.4)	3.3 (-1.0 - 7.7)	0.6 (-0.5 - +1.6)	0.89

Data presented are mean and 95% confidence interval. Significance was tested by using the paired student's *t *test under the Bonferroni-adjusted significance level of p = 0.0045 (= 0.05/11) for multiple comparisons.

The results of stimulation using collagen, which is unspecific for the evaluation of the antiplatelet effectiveness of aspirin, revealed no consistent influence of either aspirin treatment options and were therefore not included in the further analysis (Table 3).

**Table 3:** Results of collagen-induced aggregometry for the 3 treatment groups

	Aspirin 200 mg oral	Aspirin 500 mg oral	**Aspirin 500 mg i.v**.	P-value (200 mg vs. 500 mg oral)
**basal**	16.9 (14.4-19.5)	20.1 (16.0-24.1)	21.4 (19.2-23.6)	0.19

**0.5 min**	-	-	15.9 (13.4-18.4)	-

**1 min**	-	-	17.0 (14.7-19.3)	-

**2.5 min**	-	-	16.7 (14.1-19.2)	-

**5 min**	17.1 (14.5-19.7)	18.8 (15.3-22.2)	16.2 (13.4-19.0)	0.49

**7.5 min**	-	-	17.0 (12.8-21.2)	-

**10 min**	15.8 (12.2-19.5)	19.6 (15.5-23.6)	18.8 (13.2-24.4)	0.19

**15 min**	15.2 (11.3-19.0)	19.8 (16.0-23.7)	16.6 (9.1-24.1)	0.14

**30 min**	14.6 (10.5-18.7)	13.7 (9.0-18.4)	17.8 (15.1-20.5)	0.77

**45 min**	12.6 (8.6-16.6)	12.3 (7.0-17.6)	12.8 (2.6-22.9)	0.79

**60 min**	11.8 (8.3-15.2)	13.0 (7.2-18.8)	16.8 (11.7-21.9)	0.74

**120 min**	14.7 (11.0-18.4)	16.2 (10.6-21.9)	17.4 (11.9-22.6)	0.68

**180 min**	14.0 (10.7-17.3)	15.7 (6.5-24.8)	16.8 (9.8-23.8)	0.69

**240 min**	14.4 (7.7-21.1)	13.3 (3.0-23.7)	18.6 (14.2-23.0)	0.82

**Day 2**	14.8 (11.4-18.1)	14.8 (8.5-21.1)	16.0 (13.1-18.9)	0.59

Values in ohm (Ω) with mean (95% CI); significance was tested by using the paired Student's *t *test under the Bonferroni-adjusted significance level of p = 0.0045 (= 0.05/11) for multiple comparisons. vs - versus.

### Time Course of the Antiplatelet Effect of Different Aspirin Dosing Schemes

**Figure 1:**
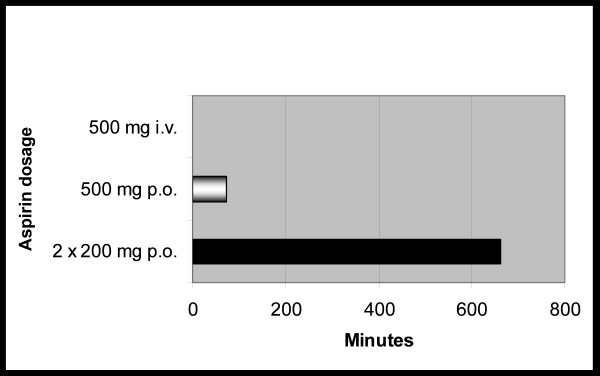
**Comparing treatment options for initiating aspirin treatment**. Mean time until the inhibitory antiplatelet effect of aspirin was evident following an aspirin dose of 500 mg intravenously (i.v.) (within 0.5 min), 500 mg aspirin tablets orally (74 ± 105 min) and when 200 mg aspirin was given on the first 2 days (662 minutes (11.2 hours) ± 1390 minutes).

**Figure 2:**
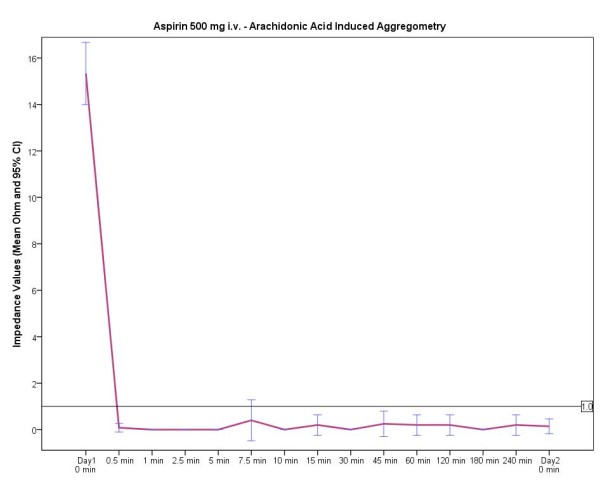
**Time course of treatment regimen 1 - 500 mg aspirin i.v. Impedance values in ohm (Ω)**. Data presented are mean and 95% confidence interval.

**Figure 3:**
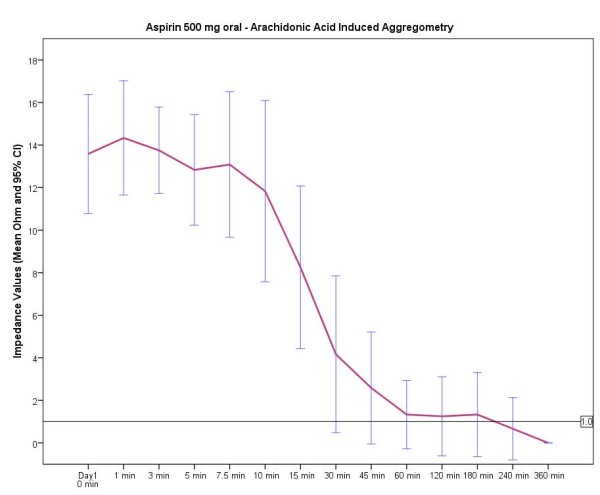
**Time course of treatment regimen 2 - 500 mg oral aspirin**. Impedance values in ohm (Ω). Data presented are mean and 95% confidence interval.

**Figure 4:**
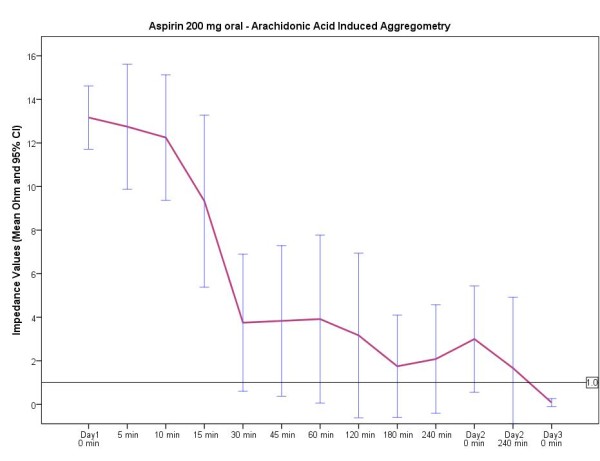
**Time course of treatment regimen 3 - 200 mg oral aspirin dosage for 2 days followed by 100 mg/d**. Impedance values in ohm (Ω). Data presented are mean and 95% confidence interval.

An overview of the mean time until the inhibitory antiplatelet effect was evident according to the different aspirin doses is provided in Figure 1. As can be seen in Figures 2, 3, 4, the curves after oral and intravenous administration differed significantly (p < 0.001 after 5 min, p = 0.04 after 10 min and p = 0.07 after 15 min respectively). Initiating antiplatelet therapy with intravenous 500 mg aspirin resulted in a consistent, sufficient and immediate platelet inhibitory response (0 Ω) within 30 seconds (Figure 2) in all 12 volunteers. When aspirin treatment according to schemes 2 and 3 was initiated, high inter- and intra-individual differences existed before gaining effective aspirin responsiveness (Figure 3 and 4). In both oral aspirin dosages, a clear decline of mean impedance values was visible at 30 min, yet not reaching sufficient antiplatelet response. The mean time until an adequate antiplatelet response was demonstrated for the treatment option of oral 500 mg aspirin was 74 min (minimum 7.5 min, maximum 360 min). Of note, in this group only 10 out of 12 volunteers had a sufficient inhibition of AA-induced platelet aggregation within 60 minutes.

**Figure 5:**
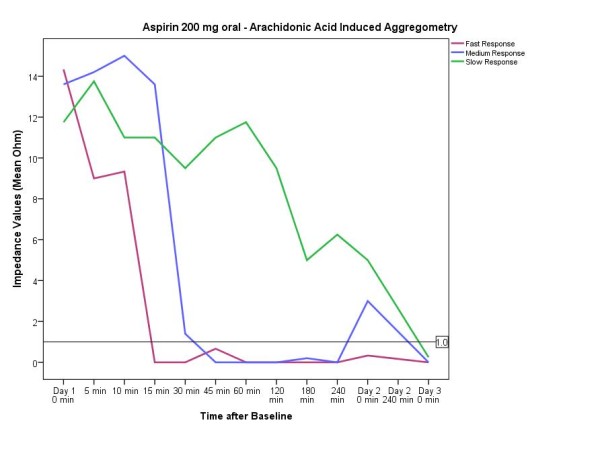
**Interindividual variability of the antiplatelet response when a loading dose of 200 mg aspirin was taken**. Impedance values in ohm (Ω). Data presented are mean and 95% confidence interval.

In the third aspirin starting scheme with two days of aspirin 200 mg (followed by aspirin 100 mg daily) the platelet inhibitory effect of aspirin was more variable. Altogether, an adequate antiplatelet effect in all participants using 200 mg aspirin orally was not detected until day 3 (mean time lag 662 min (11.2 hours); minimum 5 min, maximum 3 days). Of note, 8 out of 12 volunteers had an adequate inhibitory effect after 60 min. For this treatment group (200 mg aspirin orally) three different types of time-dependent antiplatelet effectiveness were observed: Persons with fast reactions, medium fast and slow reactions (Figure 5).

### Cessation of Aspirin Treatment - How Long Does the Antiplatelet Effect Last?

Nine volunteers were followed during the wash-out period after aspirin cessation by daily baseline controls. After the one-time oral intake of 500 mg aspirin, 3 participants showed normal aggregation results the next day, 3 on the second day and 3 volunteers on the third day. After being given 200 mg aspirin on two subsequent days followed by 100 mg daily for 5 days, 2 volunteers had normal values the day after aspirin cessation, 2 after two days, 3 after 3 days and in 2 volunteers the aspirin effect lasted 4 days.

## Discussion

Platelet aggregation profiles of our healthy participants showed a variable time response to the three different treatment dosages of aspirin in all volunteers but also revealed a high inter- and intra-individual variability among the participants. On an intravenous loading dose of 500 mg aspirin, our study indicated an immediate antiplatelet effect within 30 seconds, compared to a median time of 74 min after oral intake of 500 mg and of 11.2 hours when using 200 mg aspirin as a starting dose, respectively. These data suggest using a higher aspirin loading dose intravenously in order to achieve an early and reliable platelet inhibitory effect in acute stroke patients.

Commonly, ischemic events occurring despite aspirin therapy are qualified as therapy failures and termed as 'clinical aspirin resistance' [11]. Yet atherothrombotic complications can even occur during effective concomitant aspirin therapy when other amplification pathways play a role in platelet activation. Pathways stimulated by adenosine diphosphate (ADP), collagen, thrombin, adrenaline and sheer stress remain basically unaltered by aspirin. Therefore 'clinical aspirin resistance' should be differentiated from 'laboratory aspirin resistance', in which laboratory tests visualize the failure of aspirin to inhibit thromboxane formation entailing an insufficient inhibition of platelet function [11].

There is growing evidence that 'laboratory aspirin resistance' is of high clinical value in stroke management. Several studies measuring platelet aggregation in patients with cerebrovascular diseases were able to demonstrate different types of aspirin non-responders, a higher incidence of aspirin non-response with lower aspirin doses and enteric-coated aspirin preparations, higher recurrence rates of strokes in aspirin-nonresponsive patients and dose-dependent time intervals for recurrent events [12-16]. In this context platelet function testing allows adjustment of the antiplatelet medication in case of hypo-responsiveness and the monitoring of compliance to antiplatelet therapy [16-18].

After oral ingestion, aspirin is rapidly absorbed in the stomach and the upper intestine, reaching peak concentration in the blood within 30 to 40 min and inhibiting platelet function within one hour after intake [19]. The oral bioavailability of aspirin is approximately 40 to 50%, differing over a range of dosages as it is metabolized rapidly by esterases in the gut and in the liver before entering the systemic blood stream [20]. The intravenous injection of aspirin avoids this rapid presystemic metabolization and first-pass effect, leading to a bioavailability of up to 100%, thus enabling a more immediate antiplatelet effectiveness [21,22].

Pharmacodynamic studies on different aspirin doses for the treatment of acute stroke management are not available. Our results revealed a rapid antiplatelet effect within 30 seconds after intravenous injection of 500 mg aspirin. Orally administered aspirin in doses of 500 mg and 200 mg led to a variable inter- and intraindividual onset of action in our young healthy volunteers of up to 6 hours and 3 days respectively. Another study in normal volunteers showed that different oral preparations of 81 mg aspirin required significantly more time to reach maximum antithrombotic protection than preparations of 325 mg aspirin (day 2 and 3 versus day 1) [23]. In addition to aspirin dose and modes of administration, factors such as co-medications, gastric motility dysfunctions or gastric abnormalities in older patients or physical or emotional stress could influence the onset of the aspirin effect [24,25]. Intravenous administration of a high aspirin dosage could bypass dose- and absorption-dependent factors. A delay of aspirin response bears the disadvantage of a higher risk of recurrent cerebral ischemic events which is highest during the first days after acute cerebrovascular ischemia occurred. The estimated recurrence risk within the first 7 days after the first manifestation of TIA or minor stroke is up to 12.8% [26,27]. The therapeutic concept of the EXPRESS study contained an immediate initial therapy after diagnosis of TIA or stroke with 300 mg of aspirin followed by a prescription of either aspirin 75 mg or clopidogrel 75 mg [28]. A loading dose of 300 mg clopidogrel was recommended when clopidogrel was given. The results of this study demonstrated that the immediate treatment start after TIA was markedly effective in preventing recurrent stroke (80% risk reduction) [28]. Both the EXPRESS clinical findings and our laboratory data support the necessity to initiate antiplatelet treatment earlier and more aggressively after acute strokes.

Aspirin, an irreversible non-selective cyclooxygenase inhibitor, solely blocks one amplification pathway of platelet activation with inhibition of the thromboxane A2 synthesis. Due to the irreversible binding, this effect lasts 7 to 10 days throughout the entire life span of the platelet. Keeping this aspect in mind as well as the assumption that stopping aspirin intake will result in normal platelet function 4 to 5 days later in approximately 50% of platelets and after 7 to 10 days in more than 90%, it is recommended to stop administering aspirin 7 to 10 days prior to surgery [29]. In accordance with other working groups [23,30-32] our study demonstrated that normal baseline values in aggregometry testing were reached within 3 to 4 days after aspirin cessation. Hence, aggregometry testing to monitor the activity of antiplatelet agents could help to minimize postponement of surgical or interventional procedures in patients treated with antiplatelet agents.

All in all, new concepts for a more rapid and reliable approach for starting antiplatelet therapy of acute stroke should be sought. Aggregometry-guided control of different therapeutic regimens could assist in this process. Taking all this into consideration, we recommend carrying out a sufficiently powered clinical trial, in which different loading doses should be compared in order to investigate the optimal response to aspirin in acute stroke patients without causing an exceedingly high bleeding incidence.

Our study has several limitations. The size of our study cohort was relatively small, but all volunteers participated in all three dosing regimens to allow not only inter- but also intraindividual analysis. Furthermore our participants were healthy young persons without any co-morbidities or co-medication. However, these results of the platelet function tests on our healthy subjects cannot automatically be transferred to acute stroke patients. The pharmacokinetic and pharmacodynamic process of aspirin metabolization in stroke patients could be influenced by concomitant diseases and co-medications leading to varied drug absorption due to pathophysiological changes in the gastrointestinal tract or drug-drug interactions [24]. Additionally, platelet hyperreactivity and variations of fibrinolytic activity in acute ischemic stroke are further aspects which could alter aspirin responsiveness and the ensuing results of platelet function testing [12,17]. And finally, we cannot deduce on the basis of our results any consequences for thromboembolic and bleeding rates in acute stroke patients.

## Conclusions

Our study reveals that the antiplatelet effect differs significantly between the three different aspirin starting dosages used with a high inter- and intraindividual variability of antiplatelet response in our healthy volunteers. The results on our healthy volunteers suggest that it could be preferable to initiate acute stroke therapy with an intravenous loading dose of 500 mg aspirin in order to ensure an early platelet inhibitory effect. However, clinical outcome studies must first define the best way to initiate antiplatelet treatment with aspirin.

## Competing interests

The authors declare that they have no competing interests.

## Authors' contributions

SM and HN drafted the manuscript, recruited the volunteers, substantially supervised the data collection and were substantially involved in the study design and statistical analysis. UO carried out the platelet function studies and was substantially involved in the patient- and data collection. HE supervised the statistical analysis and helped to draft the manuscript. All authors have read and approved the final manuscript.
